# A comparison of the effectiveness of two types of deceit detection training methods in older adults

**DOI:** 10.1186/s41235-019-0178-z

**Published:** 2019-07-22

**Authors:** Jennifer Tehan Stanley, Britney A. Webster

**Affiliations:** Department of Psychology, University of Akron, Akron, OH 44325-4301 USA

**Keywords:** Deceit detection, Aging, Training, Emotion recognition, Verbal cues

## Abstract

**Background:**

In general, people are poor at detecting deception. Older adults are even worse than young adults at detecting deceit, which might make them uniquely vulnerable to certain types of financial fraud. One reason for poor deceit detection abilities is that lay theories of cues to deception are not valid. This study compared the effectiveness of two training methods to improve deceit detection among older adults: valid facial cues versus valid verbal cues to deception. Approximately 150 older adults were randomly assigned to facial training, verbal training, or a control condition. Participants completed a pre-test deceit detection task, their assigned training, and a post-test deceit detection task.

**Results:**

Both training groups significantly improved at recognizing their respectively trained cues after training. However, the facial cue training group were less accurate at detecting deception post-test compared to pre-test and the control group exhibited improved accuracy of deceit detection from pre-test to post-test.

**Conclusions:**

These results are consistent with the body of literature on deception suggesting people hover around chance accuracy, even after training. Older adults’ facial and verbal cue recognition can be improved with training, but these improvements did not translate into more accurate deceit detection, and actually hampered performance in the facial condition. Older adults showed the most benefit from sheer practice at detecting deception (in the control condition), perhaps because this condition encouraged implicit rather than explicit judgments of deception.

## Significance

Older adults are worse than young adults at detecting deception, which may a play a role in the increasing occurrence of financial exploitation of the elderly. One reason for poor deceit detection abilities is that lay theories of cues to deception are not valid. In addition, older adults are less able than young adults to accurately identify emotional facial expressions, and this hampers their ability to detect deception (e.g., shame, fear, and “duping delight”). This study involved the training of two groups of older adults on valid cues to deception: one group on valid verbal cues and one group on valid facial cues. There was also a control condition. Results showed that although the training was effective at improving the recognition of valid cues to deception, the facial group’s performance was worse after training on facial cues, but the control group’s accuracy increased from pre-test to post-test. The most interesting finding was the success of training older adults on emotion recognition, with significant improvements in accuracy. We were surprised to find that the accuracy of older adults’ deceit detection improved in the control condition, where no training was provided. We think these data have two possible implications: (1) the training may not have boosted performance on the recognition of valid cues enough to improve the accuracy of deceit detection, or (2) older adults may benefit most from following their “gut instincts” when detecting deception, such that mere practice improves performance. Future training interventions should assess whether practice with detecting deceit improves accuracy.

Despite evidence that older adults may be uniquely vulnerable to financial exploitation due to age-related differences in the accuracy of deceit detection, there is little research on ways to reduce this vulnerability. Given recent findings pointing to specific mechanisms for age differences in deceit detection (Ruffman, Murray, Halberstadt, & Vater, 2012; Stanley & Blanchard-Fields, 2008), it is surprising that no training intervention research has been conducted with older adults to improve their accuracy in deceit detection. With the increasing age of the population, research is needed on ways to improve the accuracy of deceit detection in older adults.

## Age-related differences in deceit detection and emotion recognition

A meta-analysis of the literature on deception showed that individuals are only slightly above chance at detecting deception: an average 54% accuracy was found across more than 200 studies where 50% is chance accuracy in these dichotomous truth/lie decision tasks (Bond & DePaulo, 2006). Most of the deception research is based on young adult college student samples. However, studies on aging and deception show that older adults (age 60 years plus) are even less accurate than young adults at detecting deception (Ruffman et al., 2012; Stanley & Blanchard-Fields, 2008). A primary component of many types of financial fraud against the elderly is that the perpetrator lies to the older adult. For example, in one type of scam commonly perpetrated against older adults, victims are conned into paying for unsolicited work (e.g., roof repairs, paving, or auto repairs) that is unnecessary and often poorly or partially completed. Older adults may be uniquely vulnerable to falling victim to fraudulent schemes because they are less able to determine when someone is lying.

Interestingly, age-related differences in deceit detection are largely due to age-related differences in recognizing facial expressions of emotion (Ruffman et al., 2012; Stanley & Blanchard-Fields, 2008). In numerous studies, aging researchers have found an age-related reduction in the ability to correctly identify certain facial expressions of emotion (see Ruffman, Henry, Livingstone, & Phillips, 2008 for a meta-analysis). Specifically, young adults are more accurate than older adults at identifying angry, sad, and fearful facial expressions. Key cues to deception are micro-expressions of emotion (e.g., fleeting 40–200-ms expressions of fear, shame, and “duping delight”) that leak out when a person lies - unbeknownst to the liar (Frank & Ekman, 1997, 2004). Older adults score lower than young adults on tests of emotion recognition accuracy (correctly identifying a facial expression such as fear or shame), and under certain conditions these age differences in emotion recognition statistically account for older adults’ poorer deceit detection accuracy (relative to young adults; Ruffman et al., 2012; Stanley & Blanchard-Fields, 2008). Much of the work on age differences in emotion recognition and deceit detection use stimuli with young adult targets. However, more recent research has shown that older adults exhibit an own-age bias such that they are more likely to judge an older adult to be telling the truth than a young adult (Slessor, Phillips, Ruffman, Bailey, & Insch, 2014). This suggests that the age of the target is important when examining age differences in deceit detection.

## Deception training interventions

Now that researchers have some insight into the underlying mechanisms for age-related differences in deceit detection, it is important to apply this knowledge to a training intervention aimed at improving the deceit detection accuracy of older adults. A meta-analysis of deceit detection training studies with young adults showed that training participants to identify verbal cues to deception was the most effective method of training (Hauch, Sporer, Michael, & Meissner, 2014). For example, one established method for identifying deception using verbal cues is the criteria-based content analysis (CBCA) developed by Steller and Köhnken (1989). This method identifies 19 reality criteria for analyzing statements for credibility. Subsequent training studies eliminated 5 of the original criteria because they were only relevant to children’s testimonies or were difficult to understand (Landry & Brigham, 1992). A revised list of 14 criteria have been used successfully in a training paradigm to significantly increase deceit detection accuracy, with accuracy for untrained adults - at 47% - significantly lower than accuracy for participants trained on the CBCA - 55% (see Table 1 for list of criteria). No study has investigated whether these training techniques can improve the accuracy of deceit detection in older adults. It is important to compare the effectiveness of different training approaches in an older adult sample, given some of the unique characteristics of older adults (i.e., reduced accuracy in emotion recognition).

**Table 1:** Content criteria for statement analysis (from Steller & Köhnken, 1989)

Category	Criterion	Description for present research
General characteristics	1. Logical structure	True accounts have an *inner coherence and consistency* (Undeutsch, 1984)
	2. Quantity of details	Abundant detail is impossible to fake (e.g., detailed description of the place). Do not count repetitions of the same details
Specific contents	3. Contextual embedding	Real incidents occur within the everyday relationships and happenings of life
	4. Descriptions of interactions	Describing interactions is a sign of credibility
	5. Reproduction of conversation	Dialogue of speakers is reported
	6. Unexpected complications during the incident	Surprising problems happen in real life
Peculiarities of content	7. Unusual details	Odd details are a sign of credibility
	8. Superfluous details	When someone lies they don’t think of inventing irrelevant details, but these are often told in true accounts
	9. Accounts of subjective mental state	Reports of feelings or cognitions during the event indicate credibility
	10. Attribution of perpetrator’s mental state	Reporting the affective reactions and thoughts of others is a sign of credibility
Motivation-related contents	11. Spontaneous corrections	Liars do not correct their statements
	12. Admitting lack of memory	Liars do not admit to lack of memory
	13. Raising doubts about one’s own testimony	Liars do not raise doubts about their own testimony
	14. Self-deprecation	Liars are confident

## Valid facial cues to deception

In addition to verbal cues to deception, noticing incongruent facial expressions of emotion during a lie are valid cues to deception (Frank & Ekman, 1997, 2004; ten Brinke & Porter, 2012). Because of age-related differences in the accuracy of emotion recognition (Ruffman et al., 2008), older adults may receive the greatest benefit from learning how to identify micro-expressions of emotion (e.g., fear, shame, and “duping delight”) that are valid cues to deception. Young adults’ accuracy in emotion recognition improves with training when the training includes feedback (Elfenbein, 2006). Similarly, a meta-analysis suggested the most effective means of training young adults on the more broad ability of person perception (judgment of the internal state of others) is with practice and feedback (Blanch-Hartigan, Andrzejewski, & Hill, 2012). However, it may be difficult to improve older adults’ emotion recognition accuracy through training. Across four studies, using a lifespan sample, young and middle-aged adults improved emotion recognition accuracy after brief computerized training, while older adults’ accuracy did not improve following the training (Schlegel, Vicaria, Isaacowitz, & Hall, 2017). Given that the underlying mechanism(s) responsible for age-related differences in emotion recognition are still unclear (Isaacowitz & Stanley, 2011), it is possible that these differences are not malleable (for example, they may be rooted in brain-based functional changes associated with aging such as reduced amygdala activation in response to emotional stimuli (Cacioppo, Bernston, Bechara, Tranel, & Hawkley, 2011; Mather et al., 2004)).

## Valid verbal cues to deception

If training in the accuracy of emotion recognition is not effective in older adults, it may be more fruitful to train older adults to recognize the valid verbal cues to deception such as less speaking time, more nervousness, fewer details, less logical arguments, and less cooperation (DePaulo et al., 2003; ten Brinke, Stimson, & Carney, 2014). Interestingly, many individuals’ lay theories of deceit detection rely upon invalid cues to deception (Akehurst, Köhnken, Vrij, & Bull, 1996). For example, many people believe that arm and leg movements increase during deception when in fact they decrease, and that admitting lack of memory and raising doubts increase during deception, when in fact it decreases (Akehurst et al., 1996). Furthermore, although both young and older adults associate averted gaze with lying, when actually making judgments about veracity, older adults are less likely to use direct versus averted gaze cues than young adults (Slessor et al., 2012). Thus, it is important to understand the cues that young and older adults actually use when judging veracity.

A secondary data analysis of the Stanley and Blanchard-Fields (2008) thought-listing data investigated whether there are age differences in the cues that young and older adults use to detect deception. In this study, 166 young adults (ages 18–27 years; 49% female) and 184 older adults (ages 61–83 years; 54% female) were presented with 10 videos of young men being interrogated about whether they committed a crime (all 10 claimed innocence but half were lying). Participants made a truth versus lie judgment about each target and then listed the factors that went into their decision in an open-format thought-listing task. Participants were randomly assigned to one of three between-subjects conditions that varied based on the presentation modality of the video statements: audio-visual, visual alone, or audio alone. The main finding of the published study was that young adults were better than older adults at detecting deceit in the conditions with visual information (i.e., visual and audio-visual). Older adults were better than chance when audio information was available.

To inform the design of the present study, we coded a total of 2262 responses from the thought-listing using a theory-driven and data-driven coding scheme. Two independent coders reached 78% agreement categorizing 22% of the responses. A single coder who was blind to participant age coded the remaining responses. This pilot study is the only study to examine age differences in reported cues used to detect deception. There were age differences in the frequency of mentioning *quality of argument* as a factor used to make deception judgments. A response was coded as *quality of the argument* if the participant referenced the degree of elaboration, whether the argument was convincing, clear, or specific (e.g., “The arguments were clearly presented”). Young adults mentioned using quality of argument (55%) more often than older adults (38%), *p* < .05. Using quality of argument as a cue was positively correlated with the accuracy of deceit detection (*r*_pb_ = .25, *p* < .01). This provides initial evidence that older adults are less likely than young adults to report using valid verbal cues to detect deception, such as the quality of the argument. Thus, training older adults to recognize and use the valid verbal cues to deception may be effective at improving the accuracy of their deceit detection.

## Present study

The purpose of this study was to determine (1) the efficacy of a validated emotion recognition training tool in improving the accuracy of older adults’ emotion recognition, (2) the efficacy of a brief training tool in improving the recognition of valid verbal cues to deceit, (3) whether deceit detection training can improve accuracy in deceit detection among older adults, and (4) whether training on deciphering verbal cues to deception or facial cues to deception is more effective at improving the accuracy of deceit detection among older adults.

### Hypotheses

Because the micro-expression training tool is effective at improving the accuracy of emotion recognition among young adults (Hurley, 2012), we expected the emotion recognition training to be effective at improving the accuracy of emotion recognition for older adults (Hypothesis 1 (H1))*.* Similarly, because we created the verbal cue training to mimic the emotion recognition training as closely as possible, we expected our newly developed verbal cue training would increase accurate recognition of valid verbal cues among older adults (H2). Based on findings from a meta-analysis of the literature on deceit detection training among young adult participants, which suggests a small to medium positive effect for training studies (Hauch et al., 2014) - combined with evidence that after training older adults improve their performance on a diverse set of tasks, including visual search (Becic, Boot, & Kramer, 2008) and memory (Lachman, Weaver, Bandura, Elliott, & Lewkowicz, 1992) - we expected that deceit detection training would improve deceit detection accuracy among older adults (H3).

It was difficult to predict which training method (verbal cues or emotion recognition) would be more effective for older adults. On the one hand, findings from a meta-analysis on studies of deceit detection training with young and middle-aged adults suggests that verbal cue training is more effective than nonverbal cue training for increasing accuracy of deceit detection in general (Hauch et al., 2014). Furthermore, preliminary work in my laboratory showed that older adults were less likely than young adults to report using valid verbal cues to deception (i.e., quality of argument) and the use of these cues correlated with the accuracy of deceit detection. This suggests that older adults may indeed benefit from training on identifying valid verbal cues to deception. However, robust findings from the literature on aging highlight older adults’ poorer performance at recognizing facial expressions of emotion (Ruffman et al., 2008) and the detrimental consequences of this vulnerability for detecting deception (Ruffman et al., 2012; Stanley & Blanchard-Fields, 2008). On balance, there is currently more evidence showing that older adults perform poorly on deceit detection because of less accurate emotion recognition rather than poorer usage of verbal cues. We also expected that older adults might have more room to improve on emotion recognition training than verbal cue training. Because the links between aging, emotion recognition, and accurate deceit detection are more established in the literature than those for verbal cues, we expected that the emotion recognition intervention (facial training) would be more effective at improving older adults’ accuracy for deceit detection than the verbal training (H4). However, we expected both training groups to improve more on detecting deception post-treatment than the control group: control < verbal training < facial training (H5).

## Methods

### Participants and design

The main training study employed a three-group pre-post design. A power analysis conducted in G*Power 3.0.10 (Faul, Erdfelder, Buchner, & Lang, 2009; Faul, Erdfelder, Lang, & Buchner, 2007) indicated that a sample size of 50 participants per group would be adequate for detecting a small to medium effect. Previous research on deceit detection training reported small to medium effect sizes, and many studies used sample sizes of about 50 participants (Driskell, 2012; Hauch et al., 2014). All participants had to be free from neurological/psychological diagnoses and have normal or corrected-to-normal vision. All participants were screened for dementia and completed tests of visual and hearing acuity. Participants were recruited from our existing participant database and the local community using fliers, word of mouth, and advertisements and received US$25 for their participation. Participants could not be the same individuals who participated in the validation study.

The main training study included a total of 157 participants (ages 57–87 years; mean age = 68.76 years, *SD* = 5.80). Each participant was randomly assigned to one of three experimental conditions: verbal training, facial training, or control. Participants were screened for dementia with the Modified Telephone Interview for Cognitive Status (TICS-M 15 items; Brandt, Spencer, & Folstein, 1988; Welsh, Breitner, & Magruder-Habib, 1993). Nine participants scored below the cutoff of 21 and were not included in further analysis, leaving a total of 148 participants (ages 60–87 years; mean age = 68.96, *SD* = 5.75; *n* = 51 male and 91 female participants) for analyses. The breakdown of participants by group was as follows: 51 (15 male, 36 female) participants in the facial condition, 48 participants (22 male, 29 female) in the verbal condition, and 49 participants (20 male, 29 female) in the control condition. Within each condition, participants were randomly assigned to video set A or video set B.

### Stimuli creation

To create the videos of truth/lie statements, we recruited 20 middle-aged adults (ages 40–59 years; 50% women) because the majority of fraud against the elderly is perpetrated by middle-aged adults (Burnett, Xia, Suchting, & Dyer, 2017; DeLiema, Yonashiro-Cho, Gassoumis, Yon, & Conrad, 2017). Participants (hereafter referred to as *suspects*) were asked to lie or tell the truth about their agreement on six controversial topical issues (legalizing marijuana, euthanasia, labor unions, abortion, cloning of human cells, and government healthcare). We selected lies about attitudes and opinions (rather than a theft) because these are the most common types of lies (DePaulo et al., 2003) and we wanted to avoid putting suspects and judges into unfamiliar situations. Indeed, on average, individuals tell one or two social lies per day. Suspects were randomly assigned to lie about their opinion for three topics and tell the truth about their opinion for three topics. The interrogator was blind to which topics were lies. Suspects were provided with a monetary incentive (US$20) if they could convince the experimenter that they were telling the truth on all six statements. Past research has shown that a monetary incentive is important for creating a high-stakes lies scenario to best imitate the types of situations where con-artists lie in actual fraud or other criminal situations (Frank & Ekman, 1997; Hauch et al., 2014). Suspects’ heads and shoulders were videotaped while being interrogated with a standard set of questions (e.g., When did you first develop this opinion? Who was with you when you first developed this opinion? Are you lying to me now?).

Recent work in forensic psychology highlights the importance of employing the principle of differential recall enhancement in order to elicit content that provides discernible cues to deception (Colwell, Hiscock-Anisman, & Fede, 2013). The idea is that a person who is being honest believes that their honesty is transparent and therefore they do not try to manage their impression. In addition, a person who is being honest is able to recall the actual events from memory, which takes less effort than creating information while trying to appear consistent and credible (in the case of someone lying). We employed the principle of differential recall enhancement by simply asking at the end of each interview for the suspect to “Describe in as much detail as possible everything you remember about your opinion on this topic”. This final question can highlight differences in the credibility of statements and is now recommended as a basic requirement of standard interviewing techniques by police officers (Colwell et al., 2013).

### Validation of stimuli

In order to select the best set of stimuli for the main training study, we conducted a validation study with the sets of six statements from all 20 middle-aged suspects (a total of 120 videos, which ranged from 56 s to 4 min, 20 s (mean (*M*) = 1 min, 54 s; *SD* = 49 s). The aim was to select one truth and one lie from each suspect that yielded typical performance (based on prior research), to avoid ceiling or floor effects and allow for an increase in accuracy after the training intervention. A lifespan sample of 23 men and women (18–78 years old; *M* = 53.13 years, *SD* = 18.81; 70% women) participated in the validation study. Because judging 120 videos would likely fatigue participants, each participant viewed 30 video statements in a randomized order and judged each statement as a truth or a lie. Each video was judged by 8–10 participants (with age groups equally represented). In order to select stimuli that elicited typical, near-chance accuracy detection of truth versus deception in naïve participants, we selected one truth and one lie statement from each suspect that was *closest* to 50% accuracy in the validation study. Each suspect had three truth videos and three lie videos from which to choose one truth and one lie video for the main study. For some suspects’ videos, the accuracy across their different truth or lie statements was very low; while for other suspects’ videos, the accuracy across different truth or lie statements was much higher than 50%. For example, one suspect’s three truth videos were detected with 90%, 90%, and 100% accuracy in the validation study. In this case, we chose one of the 90%-truth videos for this suspect to be included in the main study. Another suspect’s lie videos were detected with 0%, 0%, and 11% accuracy in the validation study. We selected this suspect’s 11%-accuracy lie video to be included in the main study. This yielded 20 truth-lie pairs to use pre-test and post-test (before and after the training study). The final set of videos ranged in accuracy in the validation study from 11 to 90%. For the main study pre-test and post-test, we created two sets of videos with 10 videos (5 truths and 5 lies) for pre-test and 10 videos (5 truths and 5 lies) for post-test. Each suspect was only represented once in each video set. Thus, if the “truth” statement was used in video set A from suspect 702, then the lie statement from suspect 702 was used in video set B.

These videos were then coded for the presence of valid facial cues and verbal cues to deception. To code the facial expressions of the videos, we used the Affectiva Affdex facial expression recognition engine within the iMotions Biometric Research Platform (version 7.1 software, 2018; Copenhagen, Denmark). First, two coders independently watched the playback of the videos with the video box and points of reference to identify videos with artifacts that interfered with the facial processing. One person was chewing gum and one person brought their hand to their mouth several times, which interfered with the automated coding. This left 36 videos to code for facial expressions (18 truth-lie pairs). Because we expected individuals who were lying would try to hide some of their facial expressions (Porter & ten Brinke, 2008), we used a criterion threshold of 10% to capture fleeting and partially concealed facial expressions. Ninety percent of lies in a previous study were correctly categorized as lies just based on the presence of fear or disgust (Frank & Ekman, 1997). Coding of real televised footage of emotional pleas about a missing relative found that the presence of disgust and lower face smiles were predictive of liars (ten Brinke & Porter, 2012). Based on this prior evidence, we focused on the basic facial expressions of joy, fear, and disgust, as well as the presence of smiles (or “duping delight”). We included both joy expressions and smiles, in case the duping delight only leaked through on the lower half of the face, in a smile. The sampling rate was 17 ms. For each frame, if the software reported greater than 10% confidence that the emotion was present, that frame received a 1, all else received a zero. Then we multiplied the number of frames that exceeded the threshold by 17 to obtain the total duration (in milliseconds) of each facial expression per video. Next, we divided this duration of facial expressions by the total duration of the video to obtain a percent duration of each facial expression for each video. Given that there are individual differences in the facial expressions that leak out when lying (Frank & Ekman, 2004), we examined each video pair for the strongest emotional expressions. Nine video pairs differed on smile duration, with lie videos (*M* = 8.90%, *SE* = 3.22%) containing significantly greater overall durations of smiles than truth videos (*M* = 4.98%, *SE* = 2.50%) (paired samples *t* test: *t*(8) = 2.35, *p* = .047). Five video pairs (including one of the video pairs in the smile group) differed on the duration of the facial expression of disgust, with lie videos (*M* = 1.44%, *SE* = 0.70%) containing significantly greater overall durations of facial expressions of disgust than truth videos (*M* = 0.11%, *SE* = 0.09%) (related samples Wilcoxon signed rank test, *p* = .043). This left six video pairs without clear facial expression cues differing between truth and lie. Thus, we confirmed that 13 (of 20) video pairs contained valid facial cues to deception.

The verbal content in the videos were transcribed and verified. To code the videos for valid verbal cues to deception, two coders independently coded transcripts of the 40 videos on the 14 verbal cues presented in Table 1. All cues were coded as present (1) or absent (0), except *quantity of details*, which was coded as absent (0), some (1), or a lot (2). The codes were not mutually exclusive; each transcript could be coded as having anywhere from 0 to 14 cues present. The coders first trained on transcripts from eight videos that were not selected for this study, and then coded half of the 40 transcripts from the videos used in this study (20 transcripts). Coders worked independently, and the transcripts were stripped of identifying information such as gender and veracity. There was an overall mean kappa score (κ) = 0.88 (*SD* = 0.22) across the 14 cues and the mean Z score (κ divided by the *SD* of κ) was 3.94, indicating that agreement was significantly greater than chance (because the Z score was greater than 1.96). Discrepancies were resolved with discussion and the final codes agreed upon were used to determine whether the videos contained valid verbal cues to deception. A single coder coded the final 20 transcripts, after achieving reliability with the second coder on the first 20 transcripts.

Across all 20 video pairs, McNemar’s test for repeated measures showed that truth videos (19 of the 20 truth videos) were more likely to contain logical structure than lie videos (13 of the 20 lie videos), *p* = .031. Both quantity of details and spontaneous corrections were also valid verbal cues for a subset of these video pairs (i.e., for 10 video pairs, with 3 video pairs that had both cues). The related samples Wilcoxon signed rank test was significant (*p* = .008) for quantity of details for seven video pairs, with truth videos (*M* = 1.43, *SD* = 0.53) containing greater quantity of details than lie videos (*M* = .43, *SD* = .55). For spontaneous corrections, for six video pairs, the truth videos all had spontaneous corrections whereas none of the lie videos did (McNemar’s test for repeated measures, *p* = .031).

For the dependent variables of pre and post accuracy of deceit detection, we focused on the video pairs with valid cues to deception. Each set of videos (pre-test A, post-test A, pre-test B, and post-test B) contained at least two lies and two truths with valid cues to deception in the facial, verbal, and control conditions. Videos that did not contain facial or verbal cues were excluded from the control accuracy scores. We converted number correct to percent correct for each condition (separately pre-test and post-test) as our main dependent variables.

### Procedure

Participants sat at a computer with headphones pre-test and post-test. They adjusted the volume of headphones prior to the task using a music sample. Instructions were presented on the computer. Participants were told to watch each video and then make a judgment as to whether the suspect was lying or telling the truth. They were told that anywhere from ¼ to ¾ of the people in the videos were lying. They made their response after each video by circling truth or lie on a sheet of paper. Each video was numbered 1 through 10. Participants either saw video set A or video set B. After each test (pre and post), participants rated how accurate they thought they were at judging who was lying (on a 10-point scale). They also rated how confident they felt about their judgments (on a 10-point scale). Finally, participants listed the cues they used to detect deception. The procedure was identical pre-test, which occurred prior to training, and post-test, which occurred following training. The entire study took about 2.5 h (with breaks) to complete.

#### Training

Facial training was a self-paced tutorial through the Micro-Expression Training Tool (METT; available at paulekman.com) created by Paul Ekman. This training tool includes a benchmark assessment of accuracy in identifying micro-expressions of emotion, followed by 75 min of training and practice with feedback, and then an improvement measure assesses micro-expression accuracy again after training. The emotion recognition training consisted of 3 parts: (1) a tutorial on the evidence for valid facial cues to deception, namely that fear of getting caught, shame of lying, and joy or “duping delight” leak out during deceptive statements (approximately 15 min; Frank & Ekman, 1997), (2) completing the computerized Microexpression Training Tool (eMett 3.0; created by Paul Ekman), which has been shown to improve the accuracy of microexpression identification and includes instructions on how to identify different microexpressions of emotion (e.g., fear, joy, shame; approximately 45 min) and, (3) practice items with feedback in the form of identifying the different microexpressions of emotion (approximately 20 min). These practice items of microexpressions are the same microexpressions that have been identified as valid cues to deception in videos from a mock crime scenario (Frank & Ekman, 1997, 2004).

We created the verbal training to match the facial training using the cadre of valid verbal cues from the literature including the 14 criteria of the CBCA described above (Table 1) and additional verbal cues provided in the literature (e.g., less fluency; Driskell, 2012; Hauch et al., 2014). The CBCA has shown satisfactory reliability and validity for detecting adult lies (Gödert, Gamer, Rill, & Vossel, 2005; Landry & Brigham, 1992). The training consisted of 3 parts: (1) a tutorial on the evidence for valid versus invalid verbal cues to deception (approximately 10 min), (2) detailed descriptions of valid verbal cues to deceit adapted from DePaulo et al. (2003; approximately 40 min) and Steller and Köhnken (1989), and (3) practice with sample transcripts of statements containing each of the valid verbal cues (approximately 20 min). Participants gained practice with identifying valid verbal cues to deceit and received feedback on their ability to identify these cues.

In order to provide diversity in the practice items, they were culled from two sources of truths and lies on different topics: (1) transcripts of videos of truths and lies we obtained from ten Brinke and colleagues (ten Brinke et al., 2014), and (2) videos we collected for another study. The videos of ten Brinke et al.’ (2014) consist of six individuals lying and six telling the truth about whether they stole money in a mock crime scenario where they were instructed to either steal the money or not but always claim innocence. These videos were transcribed and good and bad examples of each of the valid verbal cues to deception were used as sample items in the practice part of the training. In addition, we collected videos of 20 young (18–30 years; 50% women) and 20 older (60–85 years; 50% women) adults lying or telling the truth about six of their personal hopes and dreams (three lies and three truths from each person). These videos were transcribed and the transcripts were used as material for practice items of valid and invalid verbal cues to deception.

Participants started with a benchmark assessment of correct categorization of verbal statements to categories of valid verbal cues to deception. There were five categories: quantity of detail, contextual embedding, admitting doubt, spontaneous corrections, and self-deprecation. Next, participants worked through a self-paced tutorial where they learned the categories and received feedback on practice trials. Finally, participants completed an improvement measure to assess their accuracy at categorizing verbal cues after the training. The entire verbal training took about 75 min.

For the control condition, participants completed a series of questions on the computer presented using Qualtrics software (Qualtrics, Provo, UT). Questions included logic and math problems, personality items, perceptual items/optical illusions, and puzzles. This took about 75 min.

### Coding of cues

Participants listed the cues or strategies they used to make truth/lie judgments twice during the study: once after the deceit detection pre-test (after all 10 truth/lie judgments) and once after the deceit detection post-test (after the second set of all 10 truth/lie judgments). This yielded two thought-listing responses for each participant. Specifically, participants were asked: “What cues or strategies did you use to determine which statements were truths and which statements were lies?”

A theory and data-driven coding scheme was developed for these open-ended responses. The coding scheme included the following 14 categories: hesitation, facial expressions, eye movements, logical response, recall of comments, speech characteristics, nonverbal behavior, nervous manner, details/context, personal beliefs, liar’s use of notes, miscellaneous, no cue reported/guessing, and not codeable. Two coders independently coded 20% of the 314 responses. Inter-rater agreement was high (85–100%). Coders discussed discrepancies to reach an agreement. A single coder coded the remainder of the responses.

## Results

### Data checks

Unfortunately, despite random assignment to the three training conditions, there were significant differences in the accuracy of deceit detection between the conditions at pre-test, *F*(2, 132) = 8.56, *p* < .001, η_p_^2^ = .12, with participants in the facial condition (*M* = 63.70%, *SE* = 2.40%) outperforming the other two conditions pre-test (verbal *M* = 54.50%, *SE* = 2.20%; control *M* = 50.80%, *SE* = 2.10%) ps < .05. These differences in the accuracy of deceit detection between the facial condition and the other two conditions pre-test were not expected, and made it more difficult to detect the predicted Test Time × Condition interaction. There were no significant gender differences in the accuracy of deceit detection. There were no significant differences in the accuracy of deceit detection accuracy between the two video sets (A and B) at pre-test or post-test.

Graphical visualization of the data suggested that the change in the accuracy of detection of deceit (DeceitChange) variable is normally distributed. However, statistical tests of normality suggested that the change in the accuracy of deceit detection was not normally distributed for the facial condition. Analyses were run two ways: assuming normality and with nonparametric tests that do not assume normality. The pattern of findings was identical so tests with normality assumptions are reported.

### Training efficacy

**Fig. 1:**
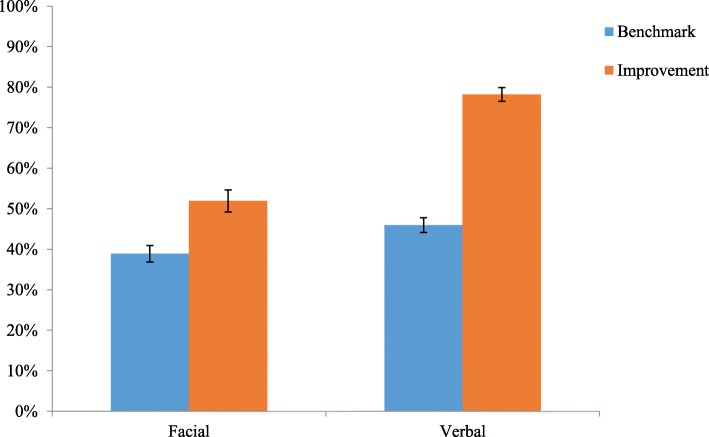
Efficacy of training. Note, bars represent standard error of the mean

For the two experimental conditions (verbal training and facial training), we assessed performance on identifying verbal categories or facial categories before training and after training. For the facial condition, participants scored *M* = 38.88%, *SD* = 14.29 for on the benchmark assessment. After the 75-min tutorial on recognizing micro-expressions, participants scored *M* = 51.94%, *SD* = 19.21, a significant improvement (*t* (49) = 6.01, *p* < .001, *d* = 0.89). Similarly, for the verbal condition, participants scored *M* = 45.95%, *SD* = 14.29 on the benchmark assessment. After the 75-min tutorial on categorizing verbal cues to categories, participants scored *M* = 78.21%, *SD* = 11.64, a significant improvement (*t* (47) = 15.89, *p* < .001, *d* = 2.30). Figure 1 depicts the results.

There was a wide range of change in performance as a function of training: For the facial condition, change in performance ranged from − 21 to 50%. For the verbal condition, change in performance ranged from 0 to 60%. All inferential analyses were conducted with only participants who scored greater than zero on the change in performance for the training: 13 participants did not meet this threshold, which left 39 participants in the facial condition, 47 in the verbal condition, and 49 in the control condition.

### Inferential statistics

**Fig. 2:**
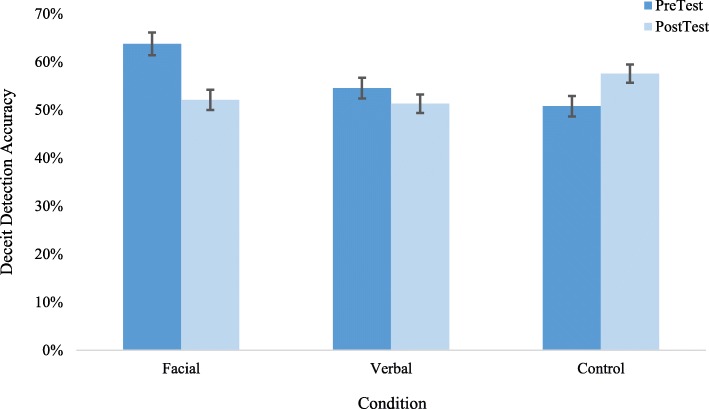
Deceit detection accuracy by condition. Note, bars are standard error of the mean

A 2 (Test time: pre versus post) × 3 (Condition: facial, verbal, control) mixed-design analysis of variance (ANOVA) was conducted on the accuracy of deceit detection with test time as a within-subjects variable. Results are depicted in Fig. 2. The Test time × Condition interaction was significant, *F*(2, 132) = 10.13, *p* < .001, η_p_^2^ = .13. Separate simple effects analyses for each condition revealed significant differences between pre-test and post-test for the facial and control conditions. For the facial condition, participants performed significantly *worse* post-test (*M* = 52.10%, *SE *= 2.10%) than pre-test (*M* = 63.7%, *SE* = 2.80%), *F* (1, 38) = 14.70, *p* < .001, η_p_^2^ = .28. For the control condition, participants performed significantly better post-test (*M *= 57.60%, *SE* = 1.80%) than pre-test (*M* = 50.80%, *SE* = 2.20%), *F* (1, 48) = 6.21, *p* = .016, η_p_^2^ = .11.

We tested whether any of the percent accuracy pre-test and post-test scores differed significantly from chance (chance = 50% in a dichotomous truth/lie decision task). The facial and verbal pre-test scores were significantly better than chance, *p*s < .01. Post-test accuracy in the control condition was significantly better than chance, *p* < .001.

### Cue analyses

Table 2 displays the frequency of cues mentioned by condition. Pilot testing of the training study indicated that it was important to inform participants that targets in the videos were told they could make notes to refer to when discussing their opinions on different topics, just as you might have notecards when giving a speech. Still, it seems that some participants factored in whether participants looked at their notes as a sign that they were lying (Table 2, “Notes”). It is also interesting that some individuals judged whether a target was lying or telling truth based on whether the target’s stated belief aligned with their own belief or not (Table 2, “Personal beliefs”). The exact McNemar’s test determined that for the facial condition, there was a significant increase in reported use of facial expressions post training relative to pre training, *p* = .031. For the verbal condition, the exact McNemar’s test determined that there was a significant decrease in eye movement cues reported pre to post training (*p* < .001) and a significant increase in the reported use of details/context (*p* = .001).

**Table 2:** Frequency of cues mentioned by condition

Cue Category	Condition					
	Facial (*N* = 39)		Verbal (*N* = 47)		Control (*N* = 49)	
	Pre-test	Post-test	Pre-test	Post-test	Pre-test	Post-test
Hesitation	21%	5%	15%	9%	8%	8%
Facial expressions	**28%**	**54%**	21%	26%	39%	29%
Eye movement	59%	59%	**75%**	**30%**	59%	51%
Logical response	10%	8%	21%	26%	16%	16%
Restating views	28%	15%	23%	23%	14%	8%
Speech characteristics	15%	26%	32%	30%	33%	16%
Nonverbal behavior	39%	44%	38%	23%	41%	35%
Nervous behavior	28%	23%	32%	13%	25%	22%
Details/context	10%	15%	**21%**	**51%**	4%	14%
Miscellaneous	5%	13%	4%	13%	14%	22%
Personal beliefs	–	–	4%	–	2%	2%
Notes	26%	8%	30%	15%	8%	10%
No cue or guessing	5%	3%	2%	6%	–	6%

*Note.* Percentages in bold font were significantly different from pre to post test, *p* < .05

To determine whether there was an association between condition and the use of certain cues after training, the post-test cue-use frequency values were tested for independence from condition using the Pearson chi-square statistic. Separate chi-square tests were run for each of the post-test cues comparing facial versus control condition and verbal versus control condition. Table 3 displays the cues that were not independent from condition, suggesting an association between condition and post-training cue usage.

**Table 3:** Significant differences between training conditions and control in percentage of participants reporting cue usage at post test

Facial versus control					
Cue category	Facial use	Control use	Number	χ^2^(1)	*p*
Facial expressions	54%	29%	88	5.79	.016
Verbal versus control					
Cue category	Verbal use	Control use	*N*	χ^2^(1)	*p*
Eye movements	30%	51%	96	4.48	.034
Restating view	23%	8%	96	4.23	.040
Details	51%	14%	96	14.84	< .001

Point-biserial correlations were tested separately for the three conditions for post-test cue usage and post-test accuracy of deceit detection, to determine the relationship between post-test cue usage and the accuracy of post-test deceit detection. For the facial condition, greater reported usage of hesitation correlated positively with the post-test accuracy of deceit detection (*r*_pb_ (*N* = 39) = .48, *p* = .002). For the verbal condition, greater reported usage of notes correlated positively with post-test accuracy of deceit detection (*r*_pb_ (*N* = 47) = .32, *p* = .029). For the control condition, greater usage of personal beliefs on the topic correlated negatively with post-test accuracy of deceit detection (*r*_pb_ (*N* = 49) = −.44, *p* = .002).

## Discussion

This study examined the effectiveness of two training methods for improving the accuracy of deceit detection among older adults. First, we developed two training methods and tested whether older adults improved in identifying facial and verbal cues post-training. Performance at accurately identifying cues post-training significantly improved in both groups, suggesting that the training was successful. Past interventions aimed at improving emotion recognition accuracy with training has failed to improve the performance of older adults (Schlegel et al., 2017), so the success of the emotion recognition training in this study is a novel finding. The effect size for improvement in the verbal training (*d* = 2.30) was much larger than the effect size for improvement in the facial training (*d* = 0.89), suggesting greater success in training on verbal cues than on facial cues. This replicates findings from a meta-analysis on deceit detection training studies with young and middle-aged adults showing that verbal cue training is more effective than nonverbal cue training for improving the accuracy of deceit detection (Hauch et al., 2014). These results extend those findings because it may be more difficult to improve emotion recognition accuracy in older adults through training than to improve verbal-cue recognition accuracy. Given baseline age differences in emotion recognition accuracy (older adults are less accurate than young adults) and verbal ability (older adults are better than young adults), it would be interesting to see if these same training differences extend to young adult samples with the training tools used in the present study. Overall, we considered the training for both groups successful, confirming hypotheses 1 and 2.

The results for improvement in deceit detection were less promising, as we found that accuracy decreased after participants received emotion recognition training in the facial condition, and accuracy increased in the control condition where participants did not receive any training. We found no difference in performance for the verbal condition from pre to post test. Despite random assignment to the three different conditions and the use of the same pre-test videos across conditions, we found significant differences in pre-test accuracy, with participants in the facial condition being more accurate than those in the other two conditions. These unequal starting points may have played a role in this pattern of findings, because the verbal and control conditions had more room for improvement than the facial condition. We expected the two training conditions to improve deceit detection accuracy relative to the control condition, with the facial training improving accuracy more than the verbal training. None of these hypotheses were supported (hypotheses 3, 4, and 5).

Taken together, the one finding of improvement was that the control condition improved in accuracy pre-test to post-test. This suggests that older adults might do better when they go with their gut-feeling rather than analyzing the facial or verbal cues to deception. Some research suggests that first impressions are more accurate than more deliberate processing, and this advantage remains intact with age (Ambady, 2010; Ambady, Bernieri, & Richeson, 2000; Krendl, Rule, & Ambady, 2014). This might also explain why older adults performed worse in the facial condition after receiving training: older adults in this condition may have switched from more heuristic processing to more analytic processing, causing their performance to suffer. Given that performance improves from pre-test to post-test in the control condition, older adults’ deception accuracy might benefit from mere exposure to lie and truth videos. It would be interesting to compare training via practice and practice with feedback to tease apart the mechanism for improvements in the control condition (i.e., implicit processing versus practice effects).

Because these results were surprising, we analyzed the open-ended responses after the pre-test and post-test, on which cues participants actually used to determine deception to try to gain more insight into the cues the participants actually used. The coding of the qualitative data revealed several important findings. First, even though participants were told that targets were encouraged to use their notes when being interrogated about their beliefs, participants still considered referring to notes as an indicator of someone lying. If this study were conducted again, it would be better to remove this misleading cue from the target videos. Second, some participants mentioned that they thought a person was telling the truth about their opinion on a controversial social issue if the target’s stated opinion was in line with their own opinion on the topic. That is, if the target’s stated opinion matched the participant’s own opinion, the participant was more likely to judge the target as telling the truth. Although this was a rare cue, it came up often enough to be included in the coding scheme. This is very interesting in light of recent sociopolitical accusations of “fake news” whereby news that is not consistent with one’s own beliefs is deemed untrue. It seems that some individuals judge truth in terms of agreement with their own views. This “way of knowing” is outside of the scientific way of knowing. Another aspect of the personal beliefs cue is that these individuals are exhibiting a lack of theory of mind. This is consistent with a meta-analysis that found that increasing age is associated with increasing difficulty with theory of mind, or the understanding that other people have different thoughts and experiences from your own (Henry, Phillips, & Bailey, 2013). Interrogating targets in a crime scenario rather than an opinion scenario might circumvent this issue in future studies.

Participants appeared to change their cue usage according to the training they received. Participants in the facial condition reported using facial expressions as a cue post-test significantly more than participants in the control condition. Similarly, participants in the verbal condition reported using eye movements significantly less and restating views and details significantly more than the control group post-test. Cue usage post-test also positively correlated with post-test accuracy of deceit detection. Participants in the facial condition who reported using hesitation were more accurate at detecting deceit post-test. And it turns out that participants in the control group who relied on agreement with their own personal beliefs as a cue to deception were *less* accurate at detecting deceit post-test, indicating that this is not a valid cue to deception. These interesting findings suggest that there may be an interaction at play where individual differences in cue usage after training interact with training type to predict deceit detection accuracy. Future work should involve the collection of a larger sample to have adequate power to probe these more complex relationships.

## Limitations and future directions

Based on the available literature, improving accuracy at identifying valid cues to deception (facial and verbal) should have led to improvements in the accuracy of deceit detection. There are many possible reasons why this was not the case. First, differences between conditions at pre-test may have created an uneven playing field for improvement across the three conditions, with some conditions having more room for improvement than others. Second, although facial recognition and verbal cue recognition improved significantly, these might not have been sufficient improvements to lead to better accuracy in the deceit detection task. Understanding the minimally sufficient “amount” of valid cue recognition for improving the accuracy of deceit detection would help in the design of studies to train to that criterion. Third, although participants improved at recognizing the respective trained cues, the extent to which they applied these cues to the post-deceit detection videos is not clear. The thought-listing cues reported by participants suggested changes in cue usage in the expected directions with training, but these changes occurred in some, but not all of the participants. Future training studies could be more explicit in instructing participants to use the trained cues to improve their post-test accuracy in deceit detection. Given that there are valid facial and verbal cues to deception, and that people can be successfully trained on the recognition of both, it would be interesting to train participants on both types of cues and examine the effect on the accuracy of deceit detection. It may be that some individuals benefit more from the facial cue training, whereas others benefit more from the verbal training.

## Conclusions

This study is consistent with past work suggesting that people are not very good at detecting deceit, even after being trained on valid cues to deception. A major contribution of this study was the success of the facial training at improving the accuracy of older adults’ emotion recognition. This was the first study to show that the accuracy of older adults’ emotion recognition can improve with training. Another contribution of this study was the verbal-cue training tool we developed, which was successful at improving participants’ ability to identify valid verbal cues to deception. While these improvements did not translate to improvements in the accuracy of deceit detection, they provide a first step toward creating a successful training intervention. The results of this study suggest that the accuracy of older adults’ deceit detection might benefit most from sheer practice with detecting deceit. In contrast with the explicit nature of identifying valid cues to deception, older adults might perform best when relying on their gut instincts, making more implicit/holistic judgments about veracity.

## Data Availability

The datasets used and/or analyzed during the current study are available from the corresponding author upon reasonable request.
